# Tightly Coupled Low Cost 3D RISS/GPS Integration Using a Mixture Particle Filter for Vehicular Navigation

**DOI:** 10.3390/s110404244

**Published:** 2011-04-08

**Authors:** Jacques Georgy, Aboelmagd Noureldin

**Affiliations:** 1 Trusted Positioning Inc., Calgary, AB, T2L 2K7, Canada; 2 Electrical and Computer Engineering Department, Royal Military College of Canada, Kingston, ON, K7K 7B4, Canada; 3 Electrical and Computer Engineering Department, Queen’s University, Kingston, ON, K7L 3N6, Canada; E-Mail: aboelmagd.noureldin@rmc.ca

**Keywords:** land vehicle navigation, inertial sensors, MEMS sensors, GPS, particle filter, Kalman filter, tightly coupled INS/GPS integration

## Abstract

Satellite navigation systems such as the global positioning system (GPS) are currently the most common technique used for land vehicle positioning. However, in GPS-denied environments, there is an interruption in the positioning information. Low-cost micro-electro mechanical system (MEMS)-based inertial sensors can be integrated with GPS and enhance the performance in denied GPS environments. The traditional technique for this integration problem is Kalman filtering (KF). Due to the inherent errors of low-cost MEMS inertial sensors and their large stochastic drifts, KF, with its linearized models, has limited capabilities in providing accurate positioning. Particle filtering (PF) was recently suggested as a nonlinear filtering technique to accommodate for arbitrary inertial sensor characteristics, motion dynamics and noise distributions. An enhanced version of PF called the Mixture PF is utilized in this study to perform tightly coupled integration of a three dimensional (3D) reduced inertial sensors system (RISS) with GPS. In this work, the RISS consists of one single-axis gyroscope and a two-axis accelerometer used together with the vehicle’s odometer to obtain 3D navigation states. These sensors are then integrated with GPS in a tightly coupled scheme. In loosely-coupled integration, at least four satellites are needed to provide acceptable GPS position and velocity updates for the integration filter. The advantage of the tightly-coupled integration is that it can provide GPS measurement update(s) even when the number of visible satellites is three or lower, thereby improving the operation of the navigation system in environments with partial blockages by providing continuous aiding to the inertial sensors even during limited GPS satellite availability. To effectively exploit the capabilities of PF, advanced modeling for the stochastic drift of the vertically aligned gyroscope is used. In order to benefit from measurement updates for such drift, which are loosely-coupled updates, a hybrid loosely/tightly coupled solution is proposed. This solution is suitable for downtown environments because of the long natural outages or degradation of GPS. The performance of the proposed 3D Navigation solution using Mixture PF for 3D RISS/GPS integration is examined by road test trajectories in a land vehicle and compared to the KF counterpart.

## 1. Introduction

Dead reckoning techniques, such as inertial navigation and odometry, are integrated with GPS to provide a navigation solution which does not suffer from interruption or degradation. Such interruptions and degradations in the positioning solution happen with GPS-only navigation in urban canyons, tunnels, or dense foliage [1]. When an inertial navigation system (INS) is integrated with GPS, the latter is used to compensate for the long-term error growth in position and velocity of the former, and the former presents a solution during GPS outages. For land vehicle navigation, low-cost inertial micro-electro mechanical system (MEMS)-based inertial sensors are preferred because of their suitable price. MEMS-based inertial measurement units (IMU) have other advantages such as small size, light weight and low power consumption. The common solution for integrating INS and GPS relies of a Kalman filter (KF) [2–4].

Despite the advantages of MEMS-based IMUs, the positioning performance of the INS degrades quickly over time because these sensors have complex stochastic errors that are difficult to model. This fact influences the performance of the MEMS-based INS/GPS navigation solution during GPS outages where severe position error growth occurs. The commonly used Linearized KF (LKF) and Extended KF (EKF) use linearized dynamic models for the navigation error states. According to [5,6], these Kalman filtering techniques suffer from divergence during outages when using low-cost MEMS-based IMU’s because their stochastic drift causes large drift of the navigation states and consequently causes the linearized models to be an unsuitable approximation; another factor that further validates the previous fact is that the traditional linear short memory length models used by KF are not the most adequate for these low-cost sensors. To enhance the performance of MEMS-based INS/GPS integration, nonlinear estimation techniques that do not require linearized dynamic models should be used. Nonlinear integration techniques like particle filtering (PF) [7] have been investigated and used for INS/GPS integration using different approaches [8–17]. Because of its ability to deal with nonlinear non-Gaussian models, PF can accommodate arbitrary sensor characteristics, motion dynamics, and noise distributions. These advantages have motivated the use of PF for INS/GPS integration.

A three dimensional (3D) navigation solution suitable for all wheeled moving platforms was proposed in [16], where a new combination of inertial sensors and odometry was suggested to mitigate several sources of errors in a MEMS-based full IMU. This system was called the 3D reduced inertial sensor system (RISS), and its advantages over a full IMU and over 2D dead reckoning techniques [18,19] were described in [16]. In this former work an enhanced version of PF called Mixture PF was used for loosely coupled 3D RISS/GPS integration. The 3D positioning capabilities even during GPS outages were demonstrated in [16]. However this former work did assume only white noise for the inertial sensors errors and did not use any models for the correlated errors, like the stochastic drift. In [17], the Mixture PF was used for a 2D navigation solution using loosely coupled 2D RISS/GPS integration, but the vertically aligned gyroscope was assumed to have both a white noise component and a stochastic drift. The capabilities of PF were exploited by using advanced models for this gyroscope drift; such models can’t be used with KF. A nonlinear system identification technique called Parallel Cascade Identification (PCI) was used to give insight on this gyroscope drift and the identified model was near linear but with very long memory length. A higher order auto-regressive (AR) model was used and gave similar results to the PCI model, while being more computationally efficient. This linear high memory length model can’t be used with KF because the state vector will have to be very large, and thus all the involved matrices will grow largely in both dimensions, making the application of this filter unrealistic. The idea used to employ such long memory length model inside the Mixture PF without augmenting the state vector (*i.e.*, without increasing its size) was described in [17]. Some previous works discussing the enhancement of the modeling of the stochastic errors of inertial sensors but still relying on the traditional models used for the KF-based solutions can be found in [20,21].

The current paper presents a complete solution that targets all the future work proposed in [16], by providing a solution based on Mixture PF for tightly coupled 3D RISS/GPS integration and using a higher order AR model for the stochastic gyroscope drift, not just the white noise assumption. This gyroscope drift model is used here for the 3D solution rather than the 2D presented in [17] and with the tightly coupled scheme as opposed to the loosely coupled scheme used in both [16] and [17].

In loosely-coupled integration, at least four satellites are needed to provide acceptable GPS position and velocity, which are used as measurement updates in the integration filter. The advantage of tightly-coupled integration is that it can provide GPS measurement updates even when the number of visible satellites is three or fewer, thereby improving the operation of the navigation system in degraded GPS environments by providing continuous aiding to the inertial sensors even during limited GPS satellite availability (like in urban areas and downtown cores). Tightly-coupled integration takes advantage of the fact that, given the present satellite-rich GPS constellation, it is very rare that all the satellites will be lost in any canyon. Therefore the tightly coupled scheme of integration uses information from the few available satellites. This is a major advantage over loosely coupled integration with INS which fails to acquire any aid from GPS and considers the situation of fewer than four satellites as a complete outage. Another benefit of working in the tightly coupled scheme is that satellites with bad measurements can be detected and rejected one by one.

In tightly-coupled integration, GPS raw data is used and is integrated with the inertial sensors. The GPS raw data used in this paper are pseudoranges and Doppler shifts. From the measured Doppler for each visible satellite, the corresponding pseudorange rate is calculated. In the update phase of the integration filter the pseudoranges and pseudorange rates are used as the measurement updates to update the position and velocity states of the vehicle. The measurement model that relates these measurements to the position and velocity states is a nonlinear model. The KF integration solutions linearize this model. PF, with its ability to deal with nonlinear models, is able to give better performance for tightly-coupled integration because it uses the exact nonlinear measurement model; this is in addition to the fact that the system model is always (in tightly or loosely coupled integration) a nonlinear model and not a linearized system model like the KF case. Thus PF is able to give a better performance than KF for tightly-coupled integration.

In this paper, and in a manner alike to what was described in [17], measurement updates for the stochastic gyroscope drift are used. These updates are derived from GPS position and velocity readings together with an unaided 3D RISS mechanization. In order to benefit from these updates and GPS-derived update for azimuth as well, which are loosely-coupled updates (since they rely on GPS position and velocity readings), in addition to the benefits of tightly-coupled integration, a hybrid loosely/tightly coupled solution is proposed in this paper. This solution is suitable for downtown environments because of the long natural outages or degradation of GPS. The longer the outage, the benefit of the advanced modeling of the gyroscope drift and its measurement update is influential as demonstrated in [17], and the better the hybrid solution as compared to the normal tightly coupled solution which will not benefit from such loosely coupled updates for the azimuth and for the gyroscope drift. This fact elucidate the need for the hybrid loosely/tightly coupled scheme and why it performs better than the tightly coupled solution in long periods with degradations or interruptions that can happen in downtown scenarios. In order to achieve this hybrid solution, a routine for automatic assessment of GPS performance and detection of degraded performance is implemented, based on which the choice of loosely or tightly coupled scheme is made. If tightly coupled scheme is chosen, each visible satellite’s pseudorange measurement is separately assessed.

To summarize the contributions of this paper, it first presents the nonlinear models for tightly coupled integration to be used by a nonlinear filter such as Mixture PF without any linearization and demonstrates the higher performance over traditional linear filtering such as KF with its linearized models. Furthermore, this paper proposes a hybrid loosely/tightly coupled 3D navigation solution that uses Mixture PF for low-cost MEMS-based 3D RISS/GPS integration, with advanced modeling of the stochastic drift of the MEMS-based gyroscope and deriving measurement updates for it from GPS when adequate. A routine for automatic assessment of GPS performance, switching between the loosely and tightly coupled schemes, and assessing separate visible satellites when tightly coupled scheme is used are some of the proposed modules to enable the work in this paper. The presented full solution is aiming at providing the best possible solution in downtown scenarios with long periods of degraded or denied GPS.

## 2. Reduced Inertial Sensor System

The 2D RISS was suggested in [22], where a navigation solution based on KF for loosely coupled 2D RISS/GPS integration was proposed with the assumption that the vehicle primarily stays in the horizontal plane, while Mixture PF for loosely coupled 2D RISS/GPS integration was proposed in [23]. 2D RISS consists of a single gyroscope vertically aligned with the body frame of the vehicle together with the vehicle odometer. The 3D RISS was first proposed in [16], where Mixture PF was used for loosely coupled 3D RISS/GPS integration.

The 3D RISS uses one gyroscope, two accelerometers and the vehicle odometer to compute a 3D position, velocity, and attitude. The accelerometers are aligned with forward and transversal axis of the vehicle body frame; a reliable model for the Earth’s gravity and an odometer are used to decouple the actual acceleration of the vehicle from the accelerometer readings, thus making them appropriate to calculate pitch and roll, respectively. This configuration obviates the need of two, relatively costly and error prone gyroscopes (the two horizontal ones). The single gyroscope aligned with the vertical axis of the vehicle body frame is used together with the pitch and roll information to obtain an accurate azimuth angle in the horizontal East-North plane that is compensated for tilt errors. The forward speed derived from the vehicle odometer together with the pitch and azimuth angles is used to calculate the East, North and vertical (Up) velocities. Consequently, the latitude, longitude and the altitude of the vehicle are determined yielding a 3D position of the vehicle. The equations of 3D RISS are fully derived and explained in [16], as well as the 3D RISS advantages over using a full-IMU for wheel-based moving platforms.

As described in [16], this reduced number of sensors is enough (*i.e.*, it has the degrees of freedoms needed) to calculate a full navigation solution for wheel-based vehicles which have odometer or speed readings. This solution does not rely on any assumption that renders it unstable or misses any vehicle motion or maneuvers. This reduced number of sensors relies only on the non-holonomic constraints on such wheel-based land vehicles, whose motion is in the forward longitudinal direction with no capability to move vertically in the vehicle-body frame or sideways.

## 3. Nonlinear Models for Tightly-Coupled Integration

There are three main observables related to GPS: pseudoranges, Doppler shift (from which pseudorange rates are calculated), and the carrier phase [24,25]. This paper uses only the first two observables. Pseudoranges are the raw ranges between satellites and receiver. A pseudorange to a certain satellite is obtained by measuring the time it takes for the GPS signal to propagate from the satellite to the receiver which is then multiplied by the speed of light. The pseudorange measurement for the *m^th^* satellite is:

(1) $$\rho^{m}=c\left(t_{r}-t_{t}\right)$$

where *ρ^m^* is the pseudorange observation from the m^th^ satellite to the receiver (in meters), *t_t_* is the transmit time, *t_r_* is the receive time, and *c* is the speed of light (in meters/sec).

The satellite and receiver clocks are not synchronized and each has an offset from the GPS system time. Despite the several errors in the pseudorange measurements, the most serious is the offset of the inexpensive clock used inside the receiver from the GPS system time.

The pseudorange measurement for the *m^th^* satellite, showing the different errors contaminating it, is given as follows:

(2) $$\rho^{m}=r^{m}+{c\delta t}_{r}-{c\delta t}_{s}+{\mathrm{cI}}^{m}+{\mathrm{cT}}^{m}+{ɛ}_{\rho }^{m}$$

where *r^m^* is the true range between the receiver antenna at the receive time *t_r_* and the satellite antenna at the transmit time *t_t_* (in meters), *δt_r_* is the receiver clock offset (in seconds), *δt_s_* is the satellite clock offset (in seconds), *I^m^* is the ionospheric delay (in seconds), *T^m^* is the troposheric delay (in seconds), ${ɛ}_{\rho }^{m}$ is the error in range due to a combination of receiver noise and other errors such as multipath effects and orbit prediction errors (in meters).

The incoming frequency at the GPS receiver is not exactly the transmitted frequency from the satellite but is shifted from the original value sent. This is called the Doppler shift and it is due to relative motion between the satellite and the receiver. The Doppler shift of the m^th^ satellite, as described in [25], is the projection of relative velocities (of satellite and receiver) onto the line of sight vector multiplied by the transmitted frequency and divided by the speed of light. It is given by:

(3) $$D^{m}=\frac{((v^{m}-v)\cdot 1^{m})L_{1}}{c}$$

where ${\text{v}}^{m}={\left[v_{x}^{m},v_{y}^{m},v_{z}^{m}\right]}^{T}$ is the *m^th^* satellite velocity in the Earth-centered Earth-fixed (ECEF) frame, **v** = [*v_x_*, *v_y_*, *v_z_*]*^T^* is the true receiver velocity in the ECEF frame, *L*_1_ is the satellite transmitted frequency, and $1^{m}=\frac{{\left[\left(x-x^{m}),(y-y^{m}),(z-z^{m}\right)\right]}^{T}}{\sqrt{{\left(x-x^{m}\right)}^{2}+{\left(y-y^{m}\right)}^{2}+{\left(z-z^{m}\right)}^{2}}}={\left[1_{x}^{m},1_{y}^{m},1_{z}^{m}\right]}^{T}$ is the true line of sight vector from the *m^th^* satellite to the receiver.

Given the measured Doppler shift, the pseudorange rate *ρ̇^m^* is calculated as follows:

(4) $${\dot{\rho }}^{m}=-\frac{D^{m}c}{L_{1}}$$

### 3.1. Nonlinear Measurement Model

After compensating for the satellite clock bias, ionospheric and tropospheric errors, we can write the corrected pseudorange as [24]:

(3) $$\rho_{c}^{m}=r^{m}+{c\delta t}_{r}+{\tilde{ɛ}}_{\rho }^{m}$$

where, ${\tilde{ɛ}}_{\rho }^{m}$ represents the total effect of residual errors. The techniques to calculate corrections for satellite clock error, ionospheric and tropospheric errors can be found in [2,24,26]. This paper uses the corrections from the commercial NovAtel GPS receivers used (described later with the experimental results), these corrections come from proprietary NovAtel algorithms built-in within their receivers.

The true geometric range from the *m^th^* satellite to the receiver is the Euclidean distance and is given as follows:

(4) $$r^{m}=\sqrt{{\left(x-x^{m}\right)}^{2}+{\left(y-y^{m}\right)}^{2}+{\left(z-z^{m}\right)}^{2}}=\left\|x-x^{m}\right\|$$

where **x** = [*x*, *y*, *z*]*^T^* is the receiver position in the ECEF frame and **x***^m^* = [*x^m^*, *y^m^*, *z^m^*]*^T^* is the position of the *m^th^* satellite at the corrected transmission time but seen in “the ECEF frame at the corrected reception time of the signal”. Satellite positions are initially calculated at the transmission time in “the ECEF frame at transmission time” and not at the ECEF frame at the time of receiving the signal. According to [24], this time difference is approximately in the range of 70–90 milliseconds, during which the Earth and the ECEF rotate, and this can cause a range error of about 10–20 meters. To correct for this fact, the satellite position at transmission time has to be represented in the ECEF frame at the reception time, not the transmission time. The equations for this correction are given in [24]. The correction can either be done before the measurement model or in the measurement model itself. In the approach followed in this paper, the satellite position correction is done before the integration filter and then passed to the filter, thus the measurement model uses the corrected position reported in the ECEF at reception time. Furthermore, the satellite position correction is done by the NovAtel receivers and their proprietary algorithms.

The details of using Ephemeris data to calculate the satellites’ positions and velocities can be found in [2,24,26]. The correction mentioned above can then be achieved.

In vector form, Equation (3) is expressed as follows:

(5) $$\rho_{c}^{m}=\left\|x-x^{m}\right\|+b_{r}+{\tilde{ɛ}}_{\rho }^{m}$$

where *b_r_* = *cδt_r_* is the error in range (in meters) due to receiver clock bias. This equation is nonlinear. The traditional techniques relying on KF linearize these equations about the pseudorange estimate obtained from the inertial sensors mechanization. The details of this operation are described in [24,26]. PF is suggested in this paper to accommodate nonlinear models, thus there is no need for linearizing this equation. The nonlinear pseudorange measurement model for M satellites visible to the receiver is:

(6) $$\left[\begin{array}{c} \rho_{c}^{1}\\ \vdots \\ \rho_{c}^{M} \end{array}\right]=\left[\begin{array}{c} \left\|x-x^{1}\right\|+b_{r}+{\tilde{ɛ}}_{\rho }^{1}\\ \vdots \\ \left\|x-x^{M}\right\|+b_{r}+{\tilde{ɛ}}_{\rho }^{M} \end{array}\right]=\left[\begin{array}{c} \sqrt{{\left(x-x^{1}\right)}^{2}+{\left(y-y^{1}\right)}^{2}+{\left(z-z^{1}\right)}^{2}}+b_{r}+{\tilde{ɛ}}_{\rho }^{1}\\ \vdots \\ \sqrt{{\left(x-x^{M}\right)}^{2}+{\left(y-y^{M}\right)}^{2}+{\left(z-z^{M}\right)}^{2}}+b_{r}+{\tilde{ɛ}}_{\rho }^{M} \end{array}\right]$$

The position state **x** here is in ECEF rectangular coordinates, so it should be transformed to Geodetic coordinates (latitude *φ*, longitude *λ*, and altitude *h*) which is part of the state vector used in the Mixture PF. The relationship between the Geodetic and Cartesian coordinates is given by:

(7) $$\left[\begin{array}{c} x\\ y\\ z \end{array}\right]=\left[\begin{array}{c} \left(R_{N}+h\right)\;\text{cos}\;\varphi \;\text{cos}\;\lambda \\ \left(R_{N}+h\right)\;\text{cos}\;\varphi \;\text{sin}\;\lambda \\ \left\{R_{N}\left(1-e^{2}\right)+h\right\}\;\text{sin}\;\varphi \end{array}\right]$$

where *R_N_* is the normal radius of curvature of the Earth’s ellipsoid and *e* is the eccentricity of the Meridian ellipse. Thus the pseudorange measurement model is:

(8) $$\left[\begin{array}{c} \rho_{c}^{1}\\ \vdots \\ \rho_{c}^{M} \end{array}\right]=\left[\begin{array}{c} \sqrt{{\left(\left(R_{N}+h\right)\;\text{cos}\;\varphi \;\text{cos}\;\lambda -x^{1}\right)}^{2}+{\left(\left(R_{N}+h\right)\;\text{cos}\;\varphi \;\text{sin}\;\lambda -y^{1}\right)}^{2}+{\left(\left\{R_{N}\left(1-e^{2}\right)+h\right\}\;\text{sin}\;\varphi -z^{1}\right)}^{2}}+b_{r}+{\tilde{ɛ}}_{\rho }^{1}\\ \vdots \\ \sqrt{{\left(\left(R_{N}+h\right)\;\text{cos}\;\varphi \;\text{cos}\;\lambda -x^{M}\right)}^{2}+{\left(\left(R_{N}+h\right)\;\text{cos}\;\varphi \;\text{sin}\;\lambda -y^{M}\right)}^{2}+{\left(\left\{R_{N}\left(1-e^{2}\right)+h\right\}\;\text{sin}\;\varphi -z^{M}\right)}^{2}}+b_{r}+{\tilde{ɛ}}_{\rho }^{M} \end{array}\right]$$

The true pseudorange rate between the *m^th^* satellite and receiver is expressed as:

(9) $${\dot{r}}^{m}=1_{x}^{m}\;(v_{x}-v_{x}^{m})+1_{y}^{m}\;(v_{y}-v_{y}^{m})+1_{z}^{m}\;(v_{z}-v_{z}^{m})$$

By differentiating Equation (2), the pseudorange rate for the *m^th^* satellite can be modeled as follows:

(10) $$\begin{array}{c} {\dot{\rho }}^{m}=1_{x}^{m}\;(v_{x}-v_{x}^{m})+1_{y}^{m}\;(v_{y}-v_{y}^{m})+1_{z}^{m}\;(v_{z}-v_{z}^{m})+c\delta {\dot{t}}_{r}+{ɛ}_{\dot{\rho }}^{m}\\ =1_{x}^{m}\;(v_{x}-v_{x}^{m})+1_{y}^{m}\;(v_{y}-v_{y}^{m})+1_{z}^{m}\;(v_{z}-v_{z}^{m})+d_{r}+{ɛ}_{\dot{\rho }}^{m} \end{array}$$

where *δṫ_r_* is the receiver clock drift (unit-less), *d_r_* is the receiver clock drift (in meters/sec), ${ɛ}_{\dot{\rho }}^{m}$ is the error in observation (in meters/sec).

This last equation is linear in velocities, but it is nonlinear in position. This can be seen by examining the expression for the line of sight unit vector above. Again, there is no need for linearization because of the nonlinear capabilities of PF. The nonlinear measurement model for pseudorange rates of M satellites, again in ECEF rectangular coordinates is:

(11) $$\left[\begin{array}{c} {\dot{\rho }}^{1}\\ \vdots \\ {\dot{\rho }}^{M} \end{array}\right]=\left[\begin{array}{c} 1_{x}^{1}\;(v_{x}-v_{x}^{1})+1_{y}^{1}\;(v_{y}-v_{y}^{1})+1_{z}^{1}\;(v_{z}-v_{z}^{1})+d_{r}+{ɛ}_{\dot{\rho }}^{1}\\ \vdots \\ 1_{x}^{M}\;(v_{x}-v_{x}^{M})+1_{y}^{M}\;(v_{y}-v_{y}^{M})+1_{z}^{M}\;(v_{z}-v_{z}^{M})+d_{r}+{ɛ}_{\dot{\rho }}^{M} \end{array}\right]$$

The velocities here are in ECEF and need to be in the local-level frame because this is part of the state vector in Mixture PF. The transformation uses the rotation matrix from the local-level frame to ECEF ( $R_{\ell }^{e}$) and is as follows:

(12) $$\left[\begin{array}{c} v_{x}\\ v_{y}\\ v_{z} \end{array}\right]=R_{\ell }^{e}\left[\begin{array}{c} v_{e}\\ v_{n}\\ v_{u} \end{array}\right]=\left[\begin{array}{ccc} -\text{sin}\;\lambda & -\text{sin}\;\varphi \;\text{cos}\;\lambda & \text{cos}\;\varphi \;\text{cos}\;\lambda \\ \text{cos}\;\lambda & -\text{sin}\;\varphi \;\text{sin}\;\lambda & \text{cos}\;\varphi \;\text{sin}\;\lambda \\ 0 & \text{cos}\;\varphi & \text{sin}\;\varphi \end{array}\right]\left[\begin{array}{c} v_{e}\\ v_{n}\\ v_{u} \end{array}\right]$$

Furthermore, the line of sight unit vector from the m^th^ satellite to receiver will be expressed as follows:

(13) $$\begin{array}{l} 1^{m}=\frac{{\left[\left(\left(R_{N}+h\right)\;\text{cos}\;\varphi \;\text{cos}\;\lambda -x^{m}\right),\left(\left(R_{N}+h\right)\;\text{cos}\;\varphi \;\text{sin}\;\lambda -y^{m}\right),\left(\left\{R_{N}\left(1-e^{2}\right)+h\right\}\text{sin}\;\varphi -z^{m}\right)\right]}^{T}}{\sqrt{{\left(\left(R_{N}+h\right)\;\text{cos}\;\varphi \;\text{cos}\;\lambda -x^{m}\right)}^{2}+{\left(\left(R_{N}+h\right)\;\text{cos}\;\varphi \;\text{sin}\;\lambda -y^{m}\right)}^{2}+{\left(\left\{R_{N}\left(1-e^{2}\right)+h\right\}\text{sin}\;\varphi -z^{m}\right)}^{2}}}\\ \;\;\;\;\;={\left[1_{x}^{m},1_{y}^{m},1_{z}^{m}\right]}^{T} \end{array}$$

The combined Equations (11), (12) and (13) constitute the nonlinear pseudorange rate measurement model for M visible satellites, while Equation (8) is the nonlinear pseudorange measurement model for the M satellites. Both these models constitute the overall nonlinear measurement model used in this paper for tightly-coupled integration using Mixture PF.

### 3.2. Augmenting the System Model

The system model (described in the following section) is augmented with two states, namely: the bias of the GPS receiver clock *b_r_* and its drift *d_r_*. These two are included as states and the state vector is augmented with these two quantities. Both of these are modeled as follows:

(14) $$\left[\begin{array}{c} {\dot{b}}_{r}\\ {\dot{d}}_{r} \end{array}\right]=\left[\begin{array}{c} d_{r}+w_{b}\\ w_{d} \end{array}\right]$$

where *w_b_* and *w_d_* are white Gaussian noise terms. In discrete form it can be written as:

(15) $$\left[\begin{array}{c} b_{r,k}\\ d_{r,k} \end{array}\right]=\left[\begin{array}{c} b_{r,k-1}+\left(d_{r,k-1}+w_{b,k-1}\right)\Delta t\\ d_{r,k-1}+w_{d,k-1}\Delta t \end{array}\right]$$

where Δ*t* is the sampling time. This model is used as part of the system model described in earlier sections.

## 4. Mixture PF for Tightly-Coupled 3D RISS/GPS Integration

As discussed in the previous section, the measurement model in the case of tightly-coupled integration is a nonlinear model that relates the GPS raw measurements (pseudorange measurements and pseudorange rates) at a time epoch *k*, **z***_k_*, to the states at time *k*, **x***_k_*, and the measurement noise **ɛ***_k_*. The nonlinear measurement model for tightly-coupled integration is in the form:

(16) $${\text{z}}_{k}=\text{h}({\text{x}}_{k},{ɛ}_{k})$$

where:

(17) $${\text{z}}_{k}={\left[\begin{array}{llllll} \rho_{k}^{1} & \cdots & \rho_{k}^{M} & {\dot{\rho }}_{k}^{1} & \cdots & {\dot{\rho }}_{k}^{M} \end{array}\right]}^{T}$$

(18) $${ɛ}_{k}={\left[\begin{array}{llllll} {\tilde{ɛ}}_{\rho ,k}^{1} & \cdots & {\tilde{ɛ}}_{\rho ,k}^{M} & {ɛ}_{\dot{\rho },k}^{1} & \cdots & {ɛ}_{\dot{\rho },k}^{M} \end{array}\right]}^{T}$$

For 3D RISS, together with modeling the stochastic drift of the vertical gyroscope using a higher order AR model [17], and with the addition of the two states for GPS receiver clock bias and drift, the state vector is:

(19) $${\text{x}}_{k}={\left[\phi_{k},\lambda_{k},h_{k},v_{k}^{f},p_{k},r_{k},A_{k},\delta \omega_{k}^{z},b_{r,k},d_{r,k}\right]}^{T}$$

where *ϕ_k_* is the latitude, *λ_k_* is the longitude, *h_k_* is the altitude, $v_{k}^{f}$ is the forward velocity, *p_k_* is the pitch angle, *r_k_* is the roll angle, *A_k_* is the azimuth angle, $\delta \omega_{k}^{z}$ is the stochastic drift of the gyroscope, *b_r,k_* is the bias of the GPS receiver clock, and *d_r,k_* is its drift.

The RISS measurements provided by the odometer, the two accelerometers and the gyroscope comprises the control input; ${\text{u}}_{k-1}={\left[\begin{array}{ccccc} v_{k-1}^{\mathrm{od}} & a_{k-1}^{\mathrm{od}} & f_{k-1}^{x} & f_{k-1}^{y} & \omega_{k-1}^{z} \end{array}\right]}^{T}$ where $v_{k-1}^{\mathrm{od}}$ is the speed derived from the vehicle odometer, $a_{k-1}^{\mathrm{od}}$ is the acceleration derived from the vehicle odometer, $f_{k-1}^{x}$ is the transversal accelerometer measurement, $f_{k-1}^{y}$ is the forward accelerometer reading, and $\omega_{k-1}^{z}$ the angular rate obtained from the vertically aligned gyroscope, respectively. The corresponding process noise associated with each of the above measurements forms the process noise vector: ${\text{w}}_{k-1}={\left[\begin{array}{ccccc} \delta v_{k-1}^{\mathrm{od}} & \delta a_{k-1}^{\mathrm{od}} & \delta f_{k-1}^{x} & \delta f_{k-1}^{y} & \delta \omega_{k-1}^{z} \end{array}\right]}^{T}$ where $\delta v_{k-1}^{\mathrm{od}}$ is the stochastic error in odometer derived speed, $\delta a_{k-1}^{\mathrm{od}}$ is the stochastic error in odometer derived acceleration, $\delta f_{k-1}^{x}$ is the stochastic bias error in transversal accelerometer, $\delta f_{k-1}^{y}$ is the stochastic bias error in the forward accelerometer, and $\delta \omega_{k-1}^{z}$ is the stochastic bias error in gyroscope reading.

Thus, the system model can be formulated as:

(20) $${\text{x}}_{k}=\left[\begin{array}{c} \varphi_{k}\\ \lambda_{k}\\ h_{k}\\ v_{k}^{f}\\ p_{k}\\ r_{k}\\ A_{k}\\ \delta \omega_{k}^{z}\\ b_{r,k}\\ d_{r,k} \end{array}\right]=\text{f}({\text{x}}_{k-1},{\text{u}}_{k-1},{\text{w}}_{k-1})=\left[\begin{array}{c} \varphi_{k-1}+\frac{v_{k-1}^{f}\text{cos}A_{k-1}\text{cos}\;p_{k-1}}{R_{M}+h_{k-1}}\Delta t\\ \lambda_{k-1}+\frac{v_{k-1}^{f}\text{sin}\;A_{k-1}\text{cos}\;p_{k-1}}{\left(R_{N}+h_{k-1}\right)\text{cos}\;\varphi_{k-1}}\Delta t\\ h_{k-1}+v_{k-1}^{f}\text{sin}\;p_{k-1}\Delta t\\ v_{k-1}^{\mathrm{od}}-\delta v_{k-1}^{\mathrm{od}}\\ {\text{sin}}^{-1}\left(\frac{\left(f_{k-1}^{y}-\delta f_{k-1}^{y})-(a_{k-1}^{\mathrm{od}}-\delta a_{k-1}^{\mathrm{od}}\right)}{g}\right)\\ -{\text{sin}}^{-1}\left(\frac{\left(f_{k-1}^{x}-\delta f_{k-1}^{x})+(v_{k-1}^{\mathrm{od}}-\delta v_{k-1}^{\mathrm{od}})(\omega_{k-1}^{z}-\delta \omega_{k-1}^{z}\right)}{g\text{cos}\;p_{k}}\right)\\ {\text{tan}}^{-1}\left(\frac{U^{E}}{U^{N}}\right)+\omega^{e}\text{sin}\;\varphi_{k-1}\Delta t+\frac{v_{k-1}^{f}\text{sin}\;A_{k-1}\text{cos}\;p_{k-1}\text{tan}\;\varphi_{k-1}}{\left(R_{N}+h_{k-1}\right)}\Delta t\\ -\sum_{n=1}^{120}\alpha_{n}\delta \omega_{k-n}^{z}+\beta_{0}\omega_{k-1}^{\delta }\\ b_{r,k-1}+\left(d_{r,k-1}+w_{b,k-1}\right)\Delta t\\ d_{r,k-1}+w_{d,k-1}\Delta t \end{array}\right]$$

where *R_M_* is the Meridian radius of curvature of the Earth’s ellipsoid, *g* is the gravity acceleration, $-\sum_{n=1}^{120}\alpha_{n}\delta \omega_{k-n}^{z}+\beta_{0}\omega_{k-1}^{\delta }$ is the higher order AR model used for the stochastic gyroscope drift [17], and

(21) $$\begin{array}{l} U^{E}=\text{sin}\;A_{k-1}\;\text{cos}\;p_{k-1}\;\text{cos}\;\gamma_{k-1}^{z}-\left(\text{cos}\;A_{k-1}\;\text{cos}\;r_{k-1}+\text{sin}\;A_{k-1}\;\text{sin}\;p_{k-1}\;\text{sin}\;r_{k-1}\right)\;\text{sin}\;\gamma_{k-1}^{z}\\ U^{N}=\text{cos}\;A_{k-1}\;\text{cos}\;p_{k-1}\;\text{cos}\;\gamma_{k-1}^{z}-\left(-\text{sin}\;A_{k-1}\;\text{cos}\;r_{k-1}+\text{cos}\;A_{k-1}\;\text{sin}\;p_{k-1}\;\text{sin}\;r_{k-1}\right)\;\text{sin}\;\gamma_{k-1}^{z} \end{array}$$

(22) $$\gamma_{k-1}^{z}=\left(\omega_{k-1}^{z}-\delta \omega_{k-1}^{z}\right)\Delta t$$

The derivation of the model for the navigation states was presented in [16]. The details of using the higher order AR model for modeling the stochastic gyroscope drift can be found in [17].

In order to relate this state to the measurement model mentioned in previous sections, the following velocity transformation from the body frame to the local-level frame is needed:

(23) $$\left[\begin{array}{l} v_{k}^{e}\\ v_{k}^{n}\\ v_{k}^{u} \end{array}\right]=R_{b,k}^{\ell }\left[\begin{array}{l} 0\\ v_{k}^{f}\\ 0 \end{array}\right]=\left[\begin{array}{l} v_{k}^{f}\;\text{sin}\;A_{k}\;\text{cos}\;p_{k}\\ v_{k}^{f}\;\text{cos}\;A_{k}\;\text{cos}\;p_{k}\\ v_{k}^{f}\;\text{sin}\;p_{k} \end{array}\right]$$

The system and measurement models are nonlinear models as was seen. There is no need to linearize them because the employed technique can deal with nonlinear models. When using KF, linearized models for the navigation error states are used, and only the first order terms of the Taylor series expansion are considered. This leads to using an error-state approach where the KF estimates the error in the navigation states not the states themselves. On the other hand, the approach used in this paper is a total-state approach not an error-state approach as there is no need for linearization. So the system and measurement models used by the integration filter are the total-state nonlinear models.

### 4.1. Mixture Particle Filter

The variant of PF used in this paper is called Mixture PF. This modified version of PF was first reported in the area of robotics in [27,28] and had further elaboration in [29]. In Robotics, the Sampling/Importance Resampling (SIR) PF used for mobile robot localization is called Monte Carlo Localization (MCL) [30], and this modified version is called MCL with planned sampling [27] or Mixture MCL [29].

Before explaining how the Mixture PF enhances the performance and make this filter more efficient, the concept of SIR PF is presented briefly. One iteration of the algorithm consists of: (i) a sampling step where new samples are generated from the old sample sets and the probabilistic system model (this step corresponds to the Bayesian filtering prediction step), (ii) a weight update step where the generated samples are weighted by using the new observation and its likelihood (this step corresponds to the Bayesian filtering update step), (iii) a resampling step that eliminate the low weight samples and duplicate the higher weight ones. In any PF, the sampling step utilizes what is called an importance density function from which new samples are generated. In the case of basic SIR PF the importance density used is the prior (*i.e.*, the probabilistic system model), which consist of the system model with process noise input.

As discussed above, in the prediction phase, the SIR PF [30,31] samples from the importance density *p* (**x***_k_* |**x**_*k*–1_^(*i*)^, **u**_*k*–1_), which does not depend on the last observation. In MEMS-based INS/GPS integration, the use of the probabilistic motion model as importance density makes the SIR PF suffers from poor performance because with more drift this importance density will not produce enough samples in regions where the true PDF is large, especially in the case of MEMS-based sensors. Because of the limitation of the SIR PF, it has to use a very large number of samples to assure good coverage of the state space (for example in the order of 1,000 samples), thus making it computationally expensive. Mixture PF is a variant of PF that aims to overcome this limitation of SIR and to use much less number of samples (in this work 100 samples are used).

As described earlier, in the SIR PF the samples are predicted from the motion model, and then the most recent observation is used to adjust the importance weights of this prediction. The idea used in this enhancement to particle filtering is to add to those samples predicted from the motion model some samples predicted from the most recent observation [29]. The importance weights of these new samples are adjusted according to the probability that they came from the previous belief of vehicle state (*i.e.*, samples of the last iteration) and the latest vehicle motion. These new samples were called planned samples [27] or samples generated from the dual of MCL [29].

These planned samples are drawn from the importance density *p* (**z***_k_* |**x***_k_*) which is the observation likelihood. These samples are consistent with the most recent observation but ignorant of the previous belief about the vehicle state *p* (**x**_*k*–1_ |*Z*_*k*–1_) (where *Z*_*k*–1_ is the set of measurements from time 0 till time *k*−1) and the motion **u**_*k*–1_. These samples are weighted using *p* (**x**_*k*_^(*i*)^ |**x**_*k*–1_^(*i*)^, **u**_*k*–1_). The version of PF that uses this type of sampling alone is known as a Likelihood PF [32].

In the version of PF used in this research and as described in [29], a number of samples (a suitably chosen proportion of the total number of samples) are drawn from *p* (**z***_k_* |**x***_k_*) and added to the samples drawn from *p* (**x***_k_* |**x**_*k*–1_^(*i*)^, **u**_*k*–1_). Samples in these two groups are weighted each with its respective weight update equation, and then the resampling is carried out. According to [29], these two importance densities have complimentary advantages and disadvantages, so their combination gives better performance. This version of PF is called Mixture PF after the name used in [29], because it samples from a mixture of importance densities instead of only one. The two types of samples from the two densities are used if there is no GPS outage; this gives better performance before GPS outages and leads to a better performance during GPS outages. Furthermore, adding the samples from observation leads to faster recovery to true position after GPS outages.

### 4.2. Hybrid Loosely/Tightly Coupled Scheme

To benefit from the superior performance of some loosely coupled updates suitable for high-drift-rate low cost MEMS-based inertial sensors integrated with GPS (namely azimuth update from GPS when adequate and update of the gyroscope drift from GPS when suitable), as well as the benefits of tightly-coupled integration, the navigation solution proposed here is a hybrid solution that takes advantage of the benefits of both loosely and tightly coupled schemes.

A module for detecting degraded GPS performance, which can happen in both rural scenarios with dense canopies or downtown scenarios due to blockages, multipath or signal reflections without a direct line of sight, is used. The odometer readings, the motion constraints on land vehicles, and the high performance of the Mixture PF 3D RISS/GPS integration solution are exploited to automatically detect degraded GPS performance which routinely occurs in urban and rural canyons. First, both the number of satellites and the dilution of precision (DOP) are used as checks for the GPS information quality. Despite these two checks, some GPS readings with degraded performance (especially because of signal reflections without direct line of sight) may still find their way to update the filter and can jeopardize its performance, so further checks have to be carried on. The first check involves assessing the horizontal position provided by the GPS receiver and makes use of speed derived from odometer or wheel encoder readings. The second check is for GPS altitude and exploits the accurate estimation of this state from the Mixture PF 3D RISS. The third and fourth checks are for azimuth angle update from GPS, the third uses vehicle speed (to assure motion) and DOP, while the fourth uses motion constraints on land vehicles. The fifth check is for providing GPS update for the stochastic gyroscope drift. This check involves vehicle speed, full stationarity, and DOP. The Azimuth and gyroscope drift update also depend on the first two position checks, so if the first two checks are not met then no updates are used.

When the availability and the quality of GPS position and velocity readings pass the assessment described above, a loosely-coupled measurement update is performed for position, velocity, azimuth, and gyroscope drift. Each update is performed according to its own quality assessment. Whenever the testing procedure detects degraded GPS performance, either because the visible satellite number falls below four or because the GPS quality examination failed, the filter switches to tightly-coupled update mode. Furthermore, the measurements from each satellite are assessed independently of those of the other satellites to check whether it is adequate to use as an update. This check again exploits the higher performance of the Mixture PF for 3D RISS/GPS integration with higher order AR modeling of the gyroscope drift, since this solution can work unaided for elongated periods with only small degradation of performance. Thus the pseudorange estimate for each visible satellite to the receiver position estimated from the prediction phase of the Mixture PF is compared to the corresponding measured pseudorange to detect degradation in individual satellites measurements (for example those because of the presence of reflections with loss of direct line-of-sight). The satellites with degraded measurements are discarded, while other satellites are used for the update.

**Figure 1.**
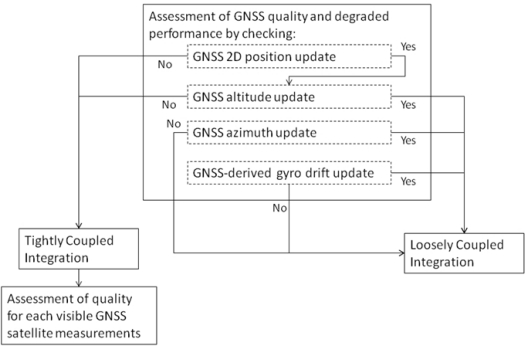
Diagram of the GNSS assessment and hybrid loosely/tightly coupled scheme choice.

The above described technique for GNSS assessment and automatic switching between loosely and tightly coupled achemes is summarized in Figure 1.

## 5. Experimental Results

The performance of the developed navigation solution is examined with road test experiments in a land vehicle. The inertial sensors used in this work are from the MEMS-grade IMU made by Crossbow, model IMU300CC-100. The specifications of this IMU are in Table 1 and the detailed specifications can be found in [33]. The forward speed derived from the vehicle built-in sensors is collected through OBD II interface using a device called CarChip, the specifications of this device are described in [34]. Some further details about speed readings through OBD II interface can be found in [35]. The results are evaluated with respect to a higher grade reference solution made by NovAtel, where Honeywell HG1700 tactical grade IMU [36] is integrated with the NovAtel OEM4 dual frequency GPS receiver. Together the NovAtel and Honeywell systems are integrated with an off-the-shelf unit developed by NovAtel, the G2 Pro-Pack SPAN unit. The details of this system are described in [37]. The NovAtel system provided the reference solution to validate the proposed method and to examine the overall performance during some intentionally introduced GPS outages. One of the presented trajectories uses the NovAtel OEM4 GPS receiver [38]; while, the other two trajectories use the NovAtel OEMV-1G GPS receiver [39], to demonstrate the performance of the proposed solution using a lower cost single frequency receiver.

Several road test trajectories were carried out using the setup described above. The sensors data were collected during the road tests and the navigation solutions were run offline using the logged data.

Three trajectories are presented here to show the performance of the proposed navigation solution in environments encompassing several different conditions. The first two have nearly open sky: (i) one with some highway sections, some rural sections, and an urban section but with open sky; (ii) the other on a highway with high speed. These two are tested with simulated partial outages. The third trajectory has downtown scenarios with frequent stops and natural GPS degradation. The first presented trajectory uses the NovAtel OEM4 GPS receiver; while, the second and third trajectories use the NovAtel OEMV-1G GPS receiver. It should be noted that both these receivers, the OEM4 and the standalone OEMV-1G provide estimates for the ionospheric delay, the tropospheric delay, and the satellite clock correction provided by NovAtel proprietary algorithms. These corrections were used to correct the pseudorange measurement before using it in the measurement model, as mentioned earlier. Furthermore these two receivers provide the corrected satellite positions at their transmission time but seen in the ECEF frame at the receive time, so no further corrections need to be implemented. These corrected satellite positions were used in the measurement model as described earlier.

The aim in the first two trajectories is to examine the performance of the proposed Mixture PF for Tightly-coupled 3D RISS/GPS integration and to compare it to KF for tightly-coupled 3D RISS/GPS integration. This is achieved by introducing simulated partial GPS outages in post-processing during portions of coverage with more than three satellites, by removing some satellites. Each of these outages is used four times with each of the two compared solutions, once with 3 satellites visible, once with 2, then 1, then 0. Having outages with 0 satellites visible is similar to what happens in loosely-coupled integration. The errors in both estimated solutions are calculated with respect to the NovAtel reference solution.

It is to be noted that this comparison is aimed at comparing the two complete navigation solutions, it is not a comparison of just two filters because the Mixture PF advantage is the capability to use the nonlinear system and measurement models without any linearization, and to use advanced models for the stochastic errors of inertial sensors that can’t be used in KF. So, it is not a comparison of two filters using the same models (it would be then all linearized models because of KF limitation), but it is a comparison of two different solutions, one of them having the ability to use better modeling. Theoretically, if the two filters were compared on the same linearized models, the PF should asymptotically converge to the KF. The PF capability to use nonlinear and advanced models is beneficial when using low cost MEMS-based inertial sensors because of the large stochastic drifts of these sensors that cause a large drift in the solution during GPS outages, which in turn cause the linearization to be around an inaccurate nominal trajectory either in EKF (closed-loop solution) or LKF (open-loop solution). This was not a noticeable problem for higher end inertial sensors in navigation and tactical grades, because there stochastic drifts were much smaller and better than low cost MEMS-based sensors.

### 5.1. First Trajectory (Open Sky with Various Road and Speed Conditions)

**Figure 2.**
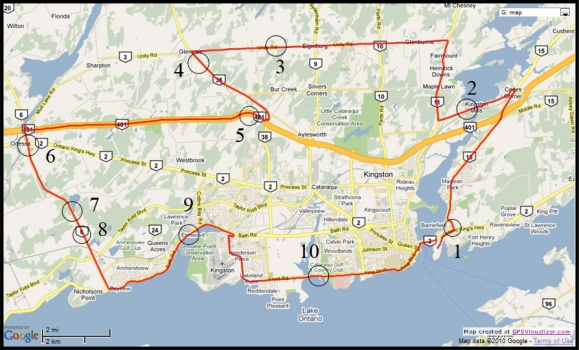
Road test trajectory around the Kingston area. Circles indicate the locations of GPS outages.

The first road test trajectory (Figure 2) is around the Kingston area in Ontario, Canada. This trajectory has some highway sections, as well as some rural and urban roadways. In addition, the terrain varies with many hills and winding turns. This road test was driven for nearly 75 min of continuous vehicle navigation at a distance of around 77 km. Ten simulated GPS outages of 60 s each (shown as circles overlaid on the map in Figure 2) were introduced such that they encompass all conditions of a typical trip including straight portions, turns, slopes, high and slow speeds. In this trajectory, the inertial sensors used for 3D RISS are from the Crossbow IMU300CC-100, the GPS receiver used is the NovAtel OEM4.

**Figure 3.**
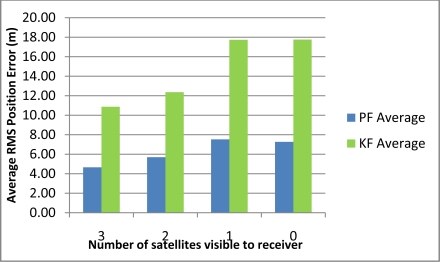
Average RMS position error over the 10 outages in Kingston trajectory.

**Figure 4.**
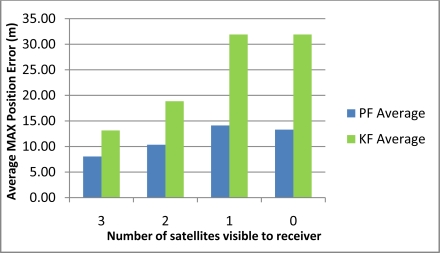
Average maximum position error over the 10 outages in Kingston trajectory.

Table 2 shows the maximum position error during the 10 simulated outages with the number of satellites varying from 3 to 0 for the two compared solutions (*i.e.*, Mixture PF with 3D RISS and KF with 3D RISS). Figures 3 and 4 illustrate the average RMS and maximum position errors, respectively, over the 10 simulated outages in each case (*i.e.*, for number of satellite visible equals 3, 2, 1, and 0).

The results in Table 2, as well as those in Figures 3 and 4, demonstrate the superiority of Mixture PF over KF in this integration problem. The main reason for this is the nonlinear capabilities of PF which enabled the use of a nonlinear system model including advanced modeling of the gyroscope drift as well as the nonlinear measurement model of the raw GPS measurements without any need for approximations during linearization. The enhancement of benefiting from more satellite availability can also be seen from these results. The general trend is that having three satellites visible is better than two, which is better than one, and which is better than the zero case. However, it should be noted that when there is only one satellite available the results are near (even sometimes worse) than the case with no satellites available. This is because of two combined reasons: (i) the good performance of the 3D RISS solution even if it works unaided for a period of time; and (ii) consequently the uncertainty added by having one satellite available is sometimes worse than the 3D RISS performance, thus it cannot provide as much aid to enhance the integrated performance but it rather sometimes make it slightly worse.

These results also show that the relative improvement of performance because of the presence of three or two satellites visible to the receiver over the scenarios where one or zero satellites are available in the case of Mixture PF is not as much as the improvement in the case of KF. This is because the 3D RISS solution with the Mixture PF and higher order AR model for the stochastic drift of the gyroscope already has a very good performance even if it works unaided (*i.e.*, the case of loosely-coupled or zero satellites visible).

**Figure 5.**
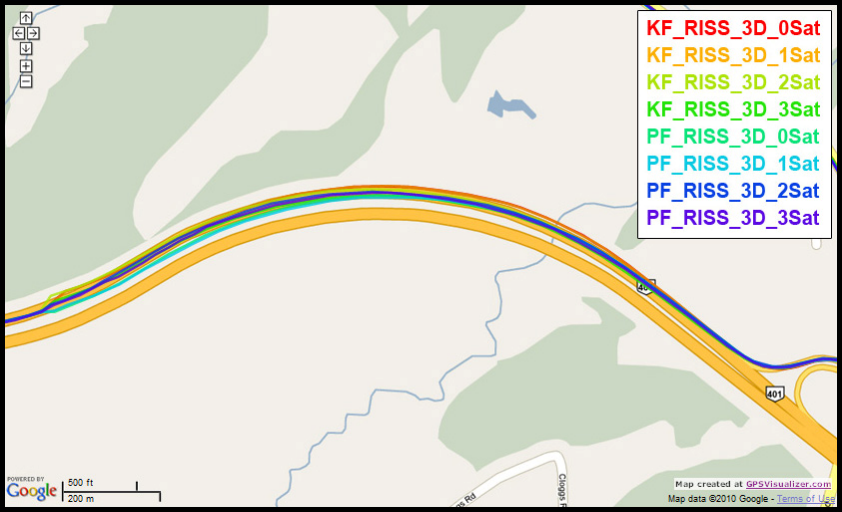
Performance during the simulated GPS outage #5 of the first trajectory.

**Figure 6.**
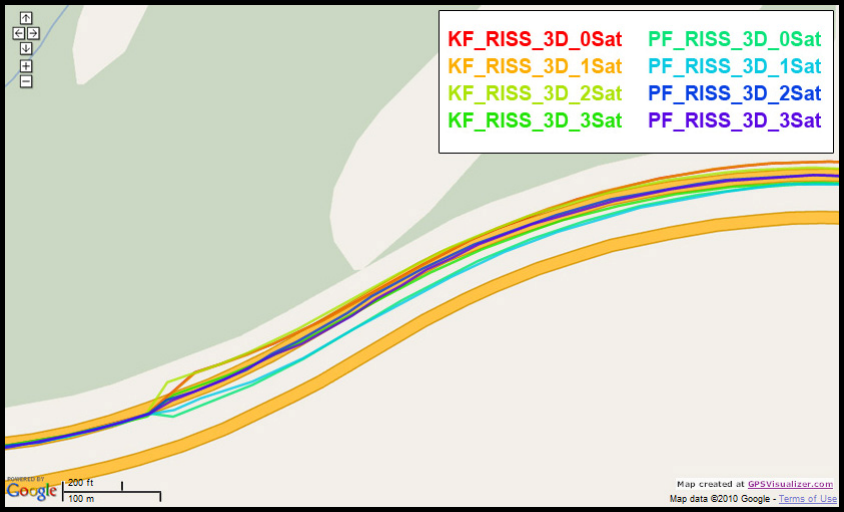
Performance towards the end of the simulated GPS outage #5 of the first trajectory.

**Figure 7.**
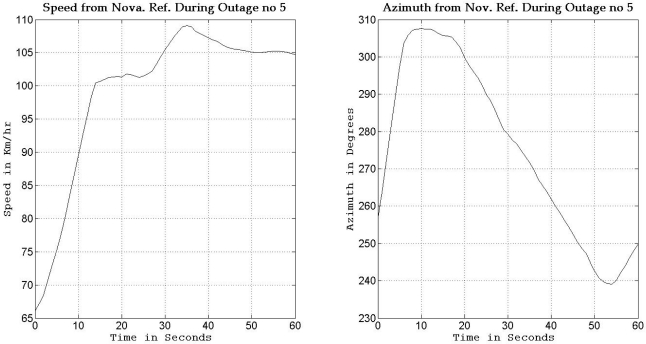
Forward speed and azimuth from Novatel reference during GPS outage #5 of the first trajectory.

**Figure 8.**
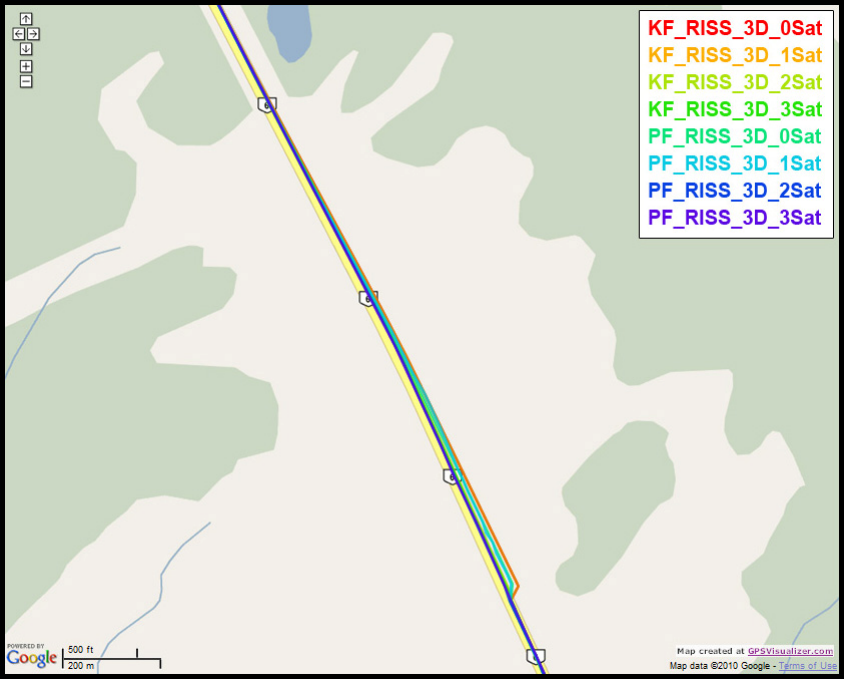
Performance during the simulated GPS outage #7 of the first trajectory.

**Figure 9.**
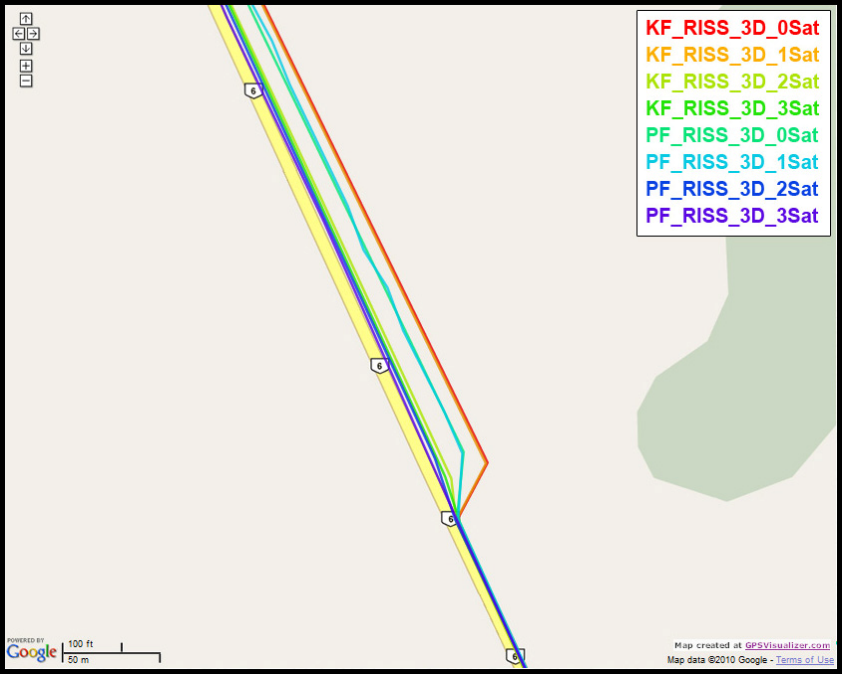
Performance towards the end of the simulated GPS outage #7 of the first trajectory.

**Figure 10.**
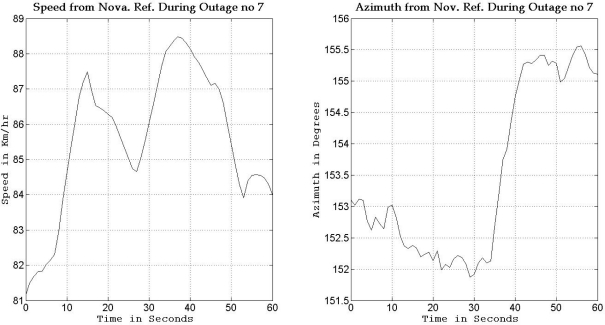
Forward speed and azimuth from Novatel reference during GPS outage #7 of the first trajectory.

To gain more insight about the performance of the two compared filters as well as the different scenarios with different numbers of satellites visible to the receiver, the details of two of these outages are discussed. Figures 5 and 8 show maps featuring the different compared solutions in the portions of the trajectory during outage numbers 5 and 7, respectively. Figures 6 and 9 provide a zoom-in on the maps towards the end of these outages, where the position error is largest as compared to the whole outage duration. To have an idea about the vehicle dynamics during these two outages, Figures 7 and 10 illustrate the forward speed of the vehicle as well as its azimuth angle both from the NovAtel reference solution for the two outages discussed.

Outage 5 is an example of an outage with turns. As can be seen from Figure 7, it has a 50° turn followed by an elongated curved road with azimuth change of about 70°. During the first turn the vehicle is accelerating from a speed of about 65 km/h to a speed of 100 km/h, during the curved highway section, the vehicle speeds vary between 100 km/h and 110 km/h. Examining the maximum position error of the different solutions during this outage, it can be seen that Mixture PF had a 10 m error when three satellites were visible, 13.1 m for two satellites, 17.8 m for one satellite, and 15.5 m for no satellites. KF had 13.75 m of error when three satellites where visible, 19.4 m for two satellites, 56.3 m for one satellite, and 57.5 m for no satellites. The KF solution during this outage was worst when one or zero satellites are visible to the receiver because of the high speed and thus longer distance traveled, and as discussed in earlier sections, any azimuth error is modulated by the speed when contributing to the position error or in other words any azimuth error will give more position error if the traversed distance is more.

Outage 7 is an example in a nearly straight road with azimuth variation of only 3° as seen in Figure 10, while the forward speed varies between 81 and 88 km/h. Examining the maximum position error of the different solutions during this outage, it can be seen that Mixture PF had a 4.9 m error when three satellites where visible, 10.3 m for two satellites, 18.5 m for one satellite, and 18.45 m for no satellites. KF had a 9.4 m error when three satellites were visible, 10.24 m for two satellites, 33.4 m for one satellite, and 33.6 m for no satellites. These results again show the benefit of having more satellites seen in a partial outage over having no satellites at all as is the case of loosely coupled integration.

### 5.2. Second Trajectory (Open Sky with High Speeds)

**Figure 11.**
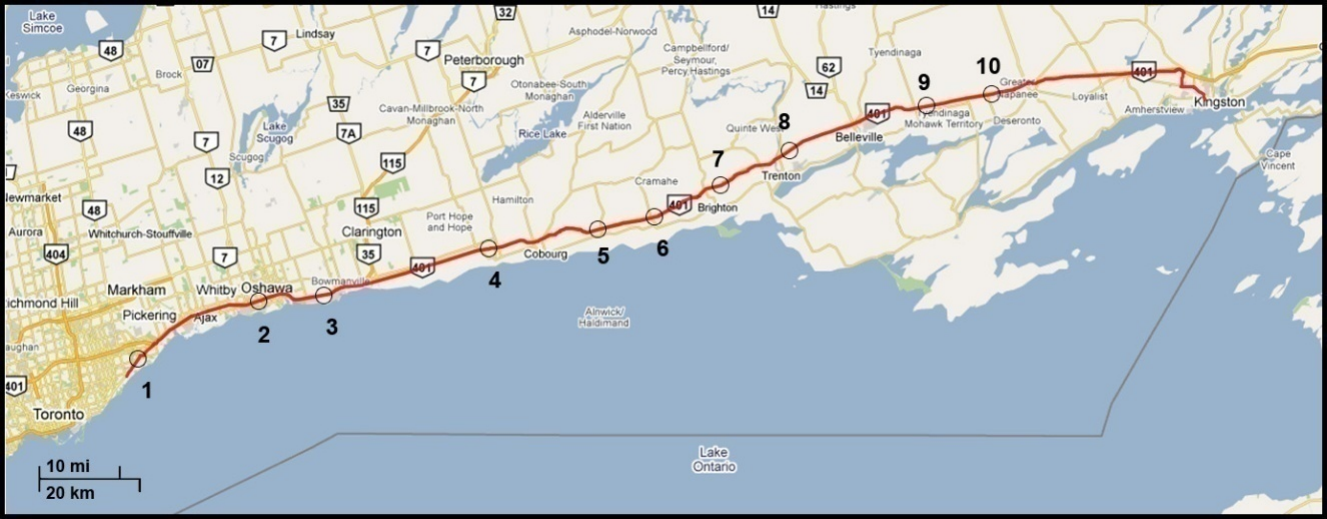
Road test trajectory from Toronto to Kingston. Circles indicate the locations of GPS outages.

The second road test trajectory (Figure 11) started in Toronto and ended in Kingston, Ontario, Canada. This trajectory had some urban roadways in Toronto, and then it continued on the highway from Toronto to Kingston. This road test was performed for nearly 140 m of continuous vehicle navigation and covered a distance of around 230 km. Ten 60-second simulated GPS outages were used (shown as circles overlaid on the map in Figure 11). The majority of the simulated outages are at high speeds. As mentioned earlier, the experiments with high speed show the robustness of the proposed solutions because higher speeds will cause more position errors due to azimuth errors.

In this trajectory, the inertial sensors used for 3D RISS were from the Crossbow IMU300CC-100 and the GPS receiver used was the NovAtel OEMV-1G. This receiver tracks both GPS and GLONASS satellites, but the work presented in this paper used only the GPS satellites. It should be noted that the drop in the number of satellites visible to the receiver, that happened several times but for very short durations, is because this highway is crossed at several points by overpasses.

**Figure 12.**
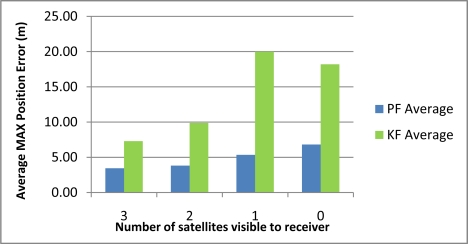
Average RMS position error over the 10 outages in Toronto-Kingston trajectory.

**Figure 13.**
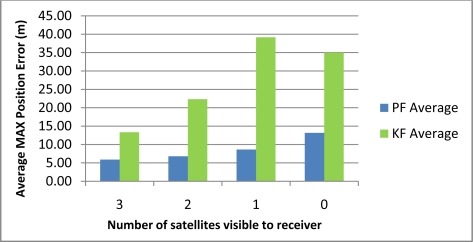
Average maximum position error over the 10 outages in Toronto-Kingston trajectory.

Table 3 shows the maximum position error during the 10 simulated outages with the number of satellites varying from 3 to 1 for the two compared solutions (*i.e.*, Mixture PF with 3D RISS and KF with 3D RISS). Figures 12 and 13 illustrate the average RMS and maximum position errors, respectively, over the 10 simulated outages in each case (*i.e.*, for number of satellites visible equal to 3, 2, 1, and 0).

The results in Table 3, as well as those in Figures 12 and 13, confirm the results of the first trajectory and demonstrate the advantages of Mixture PF over KF in this integration problem. As mentioned earlier, the main advantage is the nonlinear capabilities of PF which enabled the use of the nonlinear system model with advanced modeling of the gyroscope drift in addition to the nonlinear measurement model of the raw GPS measurements without any need for linearization. The enhancement by having more satellite availability can again be seen from these results. The general trend is that having more satellites during the partial GPS outages is better, except when there is only one satellite available the results are comparable or worse than the case with no satellites available. As discussed earlier, this is due to the good performance of 3D RISS solution even if it works totally unaided for a while which consequently causes the uncertainty added by having one satellite available being sometimes worse than the 3D RISS performance.

**Figure 14.**
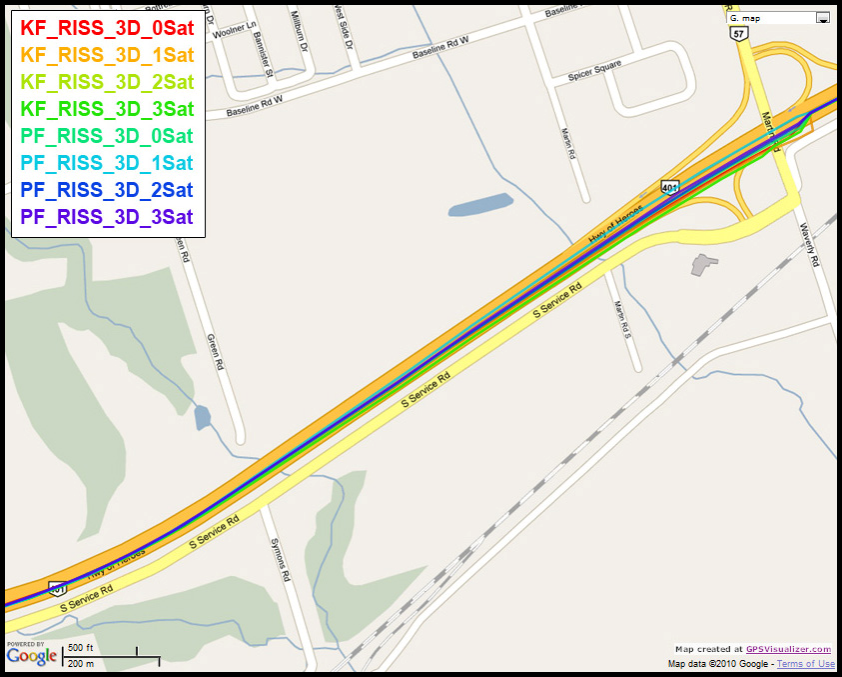
Performance during the simulated GPS outage #3 of the second trajectory.

**Figure 15.**
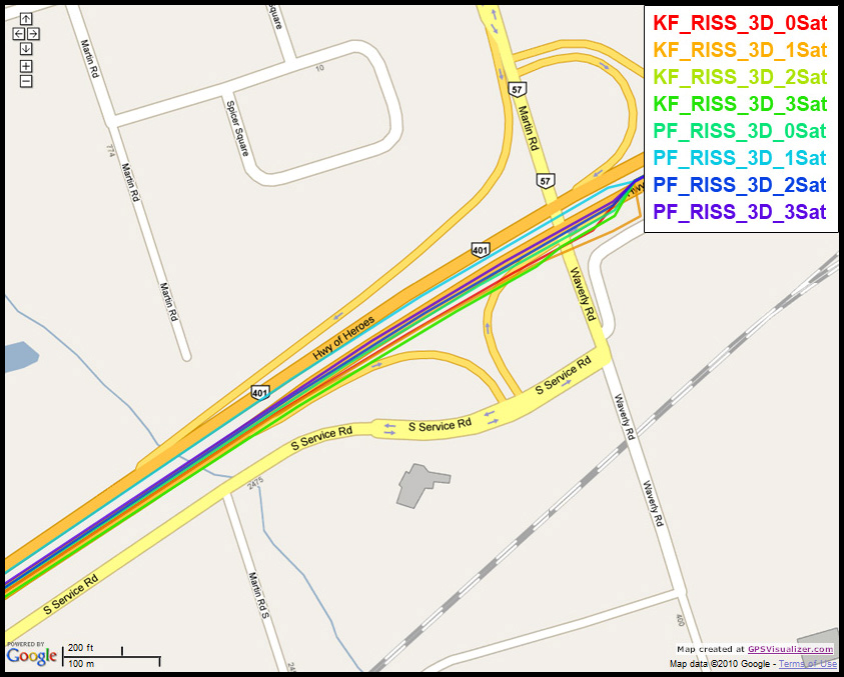
Performance towards the end of the simulated GPS outage #3 of the second trajectory.

**Figure 16.**
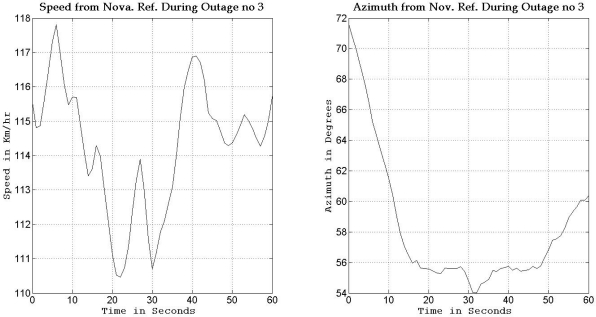
Forward speed and azimuth from Novatel reference during GPS outage #3 of the second trajectory.

To gain more insight into the performance of the compared results, outage number 3 is discussed. Figure 14 shows the map featuring the different compared solutions during outage number 3, while Figure 15 provides a zoom-in on the map towards the end of this outage, where the position error is the largest. To have an idea about the vehicle dynamics during this outage, Figure 16 shows the forward speed of the vehicle as well as its azimuth angle from the NovAtel reference solution.

Outage 3 starts with a slight turn of about 16° and then continues on a near straight portion with a deviation of 4° towards its end. The vehicle forward speed during this outage is between 111 km/hr and 117 km/h. It can be seen from the maximum position error results, that Mixture PF had a 7.9 m error when three satellites were visible, 11.25 m for two satellites, 11.4 m for one satellite, and 16.07 m for no satellites. KF gave 27.25 m of error when three satellites where visible, 27.18 m for two satellites, 45.9 m for one satellite, and 37.3 m for no satellites. The superiority of Mixture PF performance can be seen from these results.

### 5.3. Third Trajectory (Downtown Environment with Severely Degraded GPS Performance)

**Figure 17.**
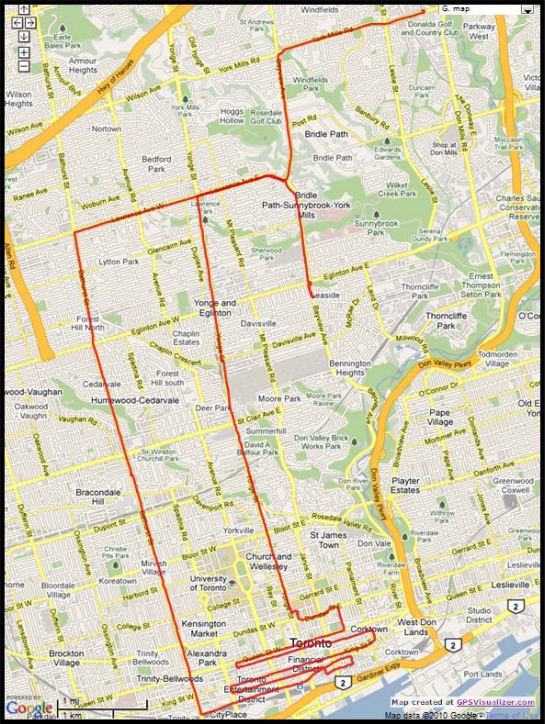
Road test trajectory in Toronto.

**Figure 18.**
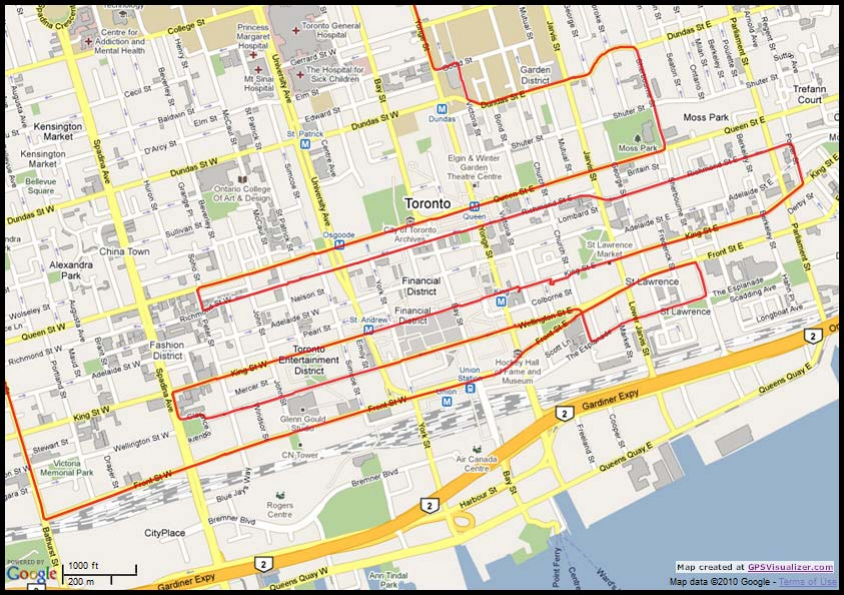
Zoom in on the downtown portion of Toronto trajectory.

The road test trajectory in downtown Toronto, Ontario, Canada presented here can be seen in Figure 17. This road test was performed for nearly 158 m of continuous vehicle navigation and a distance of around 43.8 km was traveled. This trajectory, which is in a downtown scenario with urban canyons in some parts (this part of the trajectory is shown in Figure 18), has a lot of degraded GPS performance because of either multipath, reflections with loss of direct line-of-sight, or complete blockage. The portions with degraded GPS performance encompass straight portions, turns, and frequent stops.

**Figure 19.**
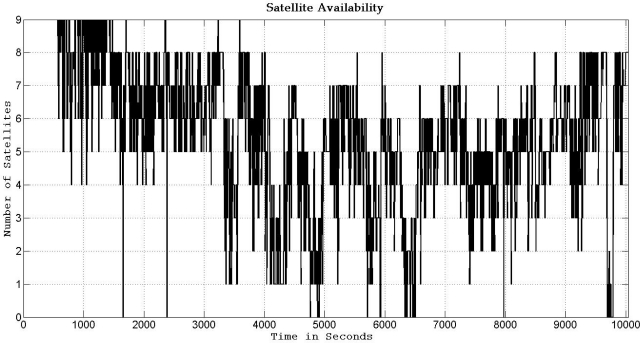
Number of GPS-only satellites visible to the NovAtel OEMV-1G receiver during the Toronto trajectory.

In this trajectory, the inertial sensors used for 3D RISS are from the Crossbow IMU300CC-100, the GPS receiver used is the NovAtel OEMV-1G. The number of the GPS-only satellites visible to the receiver over the whole trajectory duration is illustrated in Figure 19. Even though the availability of the total number of satellites visible to the receiver does not seem to be very bad, these readings are contaminated with severe effects of reflections with loss of direct line-of-sight in the urban canyons. The specific satellites with bad measurements are detected by the checking routine, as mentioned earlier, and they are rejected from being used to update the filter. Furthermore, as the work presented in this paper used only the GPS satellites (not the GLONASS satellites), thus the availability of satellites is not very high in canyons in the downtown area.

**Figure 20.**
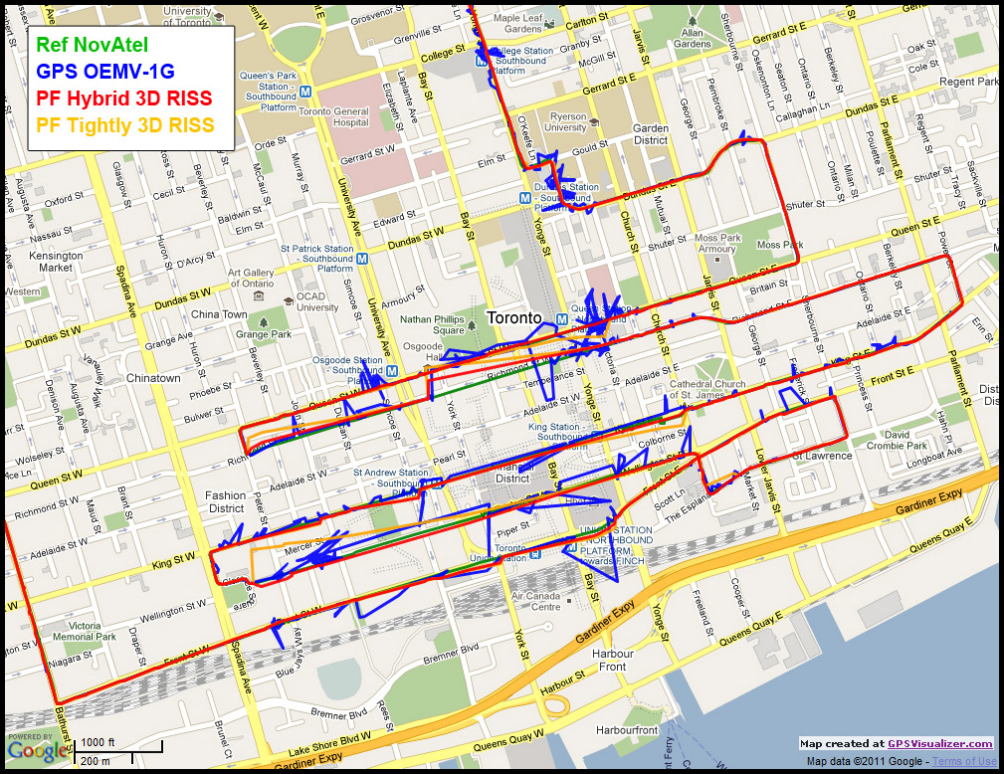
Zoom in on the downtown portion of Toronto trajectory showing the degraded GPS performance and the performance of the proposed navigation solution.

Figure 20 shows the GPS with its degraded performance, the reference solution, and the proposed navigation solution using Mixture PF for hybrid loosely/tightly coupled 3D RISS/GPS integration with higher order AR modeling of the gyroscope stochastic drift, GPS-derived updates for this drift, and automatic detection of GPS degraded performance as well as rejection of individual satellites when working in tightly-coupled mode. For the sake of comparison and demonstration of the benefit of the hybrid scheme over the normal tightly coupled scheme during long GPS outages/degradation, another solution is presented it uses Mixture PF for basic tightly coupled 3D RISS/GPS integration with automatic assessment and rejection of individual satellites but without the gyroscope drift update as it is a loosely coupled update not a tightly coupled update.

**Figure 21.**
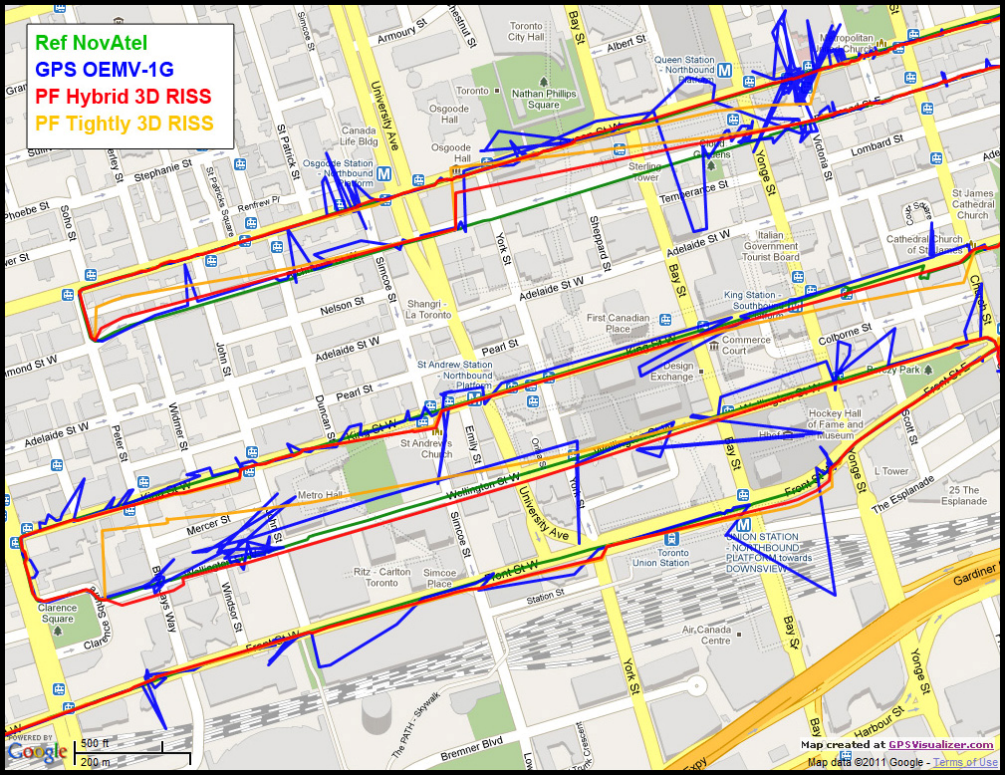
More detailed view on the downtown portion of Toronto trajectory showing the degraded GPS performance and the performance of the proposed navigation solution.

Since the trajectory had a huge number of natural GPS outages (partial or complete), Table 4 shows only the RMS and maximum position error and the RMS azimuth error during the long natural outages whose duration exceeds 100 seconds for the proposed hybrid Mixture PF 3D RISS/GPS integration solution and the basic tightly coupled Mixture PF 3D RISS/GPS. There are a large number of smaller natural outages, but for the readability of the results only the longer ones are presented. The performance during these worst outages in the trajectory can be seen in Figure 21. These results show the performance of the proposed navigation solution in a harsh environment with degraded GPS performance in deep urban canyons because of either severe effect of reflections with loss of direct line-of-sight or complete blockage. For the proposed hybrid solution, there was only one outage (outage number 3 in Table 4) that showed an unusual performance worse than all the others; it can be seen in the upper half of Figure 21. But still all these results are excellent for low cost MEMS-based inertial sensors integrated with GPS and the benefits of the hybrid solution in achieving better heading and thus positioning performance over the normal tightly coupled scheme is elucidated. The hybrid solution has the advantage of benefiting from loosely coupled type of updates which benefits the solution by providing better estimates of the stochastic drift of the gyroscope, consequently better estimates of the heading, and consequently better estimates of position. This is clear in the performance during long GPS outages (denied/degraded GPS) which are routinely encountered in downtown scenarios.

The RMS errors during the whole trajectory for pitch and roll are 0.77° and 0.29°, respectively. For the long natural outages whose duration exceeds 100 s, the RMS errors during each outage for pitch and roll are presented in Table 5. These values demonstrate the benefit of non-drifting pitch and roll estimation from accelerometers and odometer in 3D RISS, and show that this estimation is not influenced even in long GPS outages. The pitch and roll estimates in the 3D RISS is not affected by using the hybrid loosely/tightly coupled Mixture PF 3D RISS/GPS or the normal tightly coupled Mixture PF 3D RISS/GPS.

## 6. Conclusions

This paper presented a navigation solution using Mixture PF for 3D RISS/GPS integration with a higher order AR model for the stochastic drift of the vertical gyroscope as well as the proposed update for this drift from GPS when adequate, and tightly-coupled integration of 3D RISS with raw GPS measurements. The proposed solution is a hybrid loosely-coupled/tightly-coupled solution that takes advantage of the benefits of both integration schemes. As described earlier the 3D RISS consists of the vehicle odometer, a single vertically aligned gyroscope, and two horizontally aligned accelerometers.

The proposed navigation solution was tested with real road-test trajectories. The results for three trajectories were presented. The first two trajectories are open sky and 10 simulated GPS partial outages of 60-second duration were introduced in each trajectory. This was repeated four different times for each trajectory with intentionally limiting the satellites availability once to 3 satellites visible, once to 2 satellites, 1 satellite, and 0 satellites. The proposed solution based on Mixture PF was compared to a KF-based solution for the same integration problem. The third trajectory is a downtown scenario with natural GPS degradation because of multipath and complete blockage, where the Mixture PF solution was tested to demonstrate its performance in such harsh scenarios that can be met in deep urban canyons in downtown environments.

For the first two trajectories, considering the average maximum error in position, the Mixture PF solution achieved 47% improvement over KF when three satellites are visible to the receiver, 57% improvement when two satellites are visible, 67% improvement when one satellite is visible, and 60% improvement when no satellites were visible (*i.e.*, the loosely-coupled scenario).

The results showed that the proposed navigation solution using Mixture PF outperformed its KF counterpart and showed good performance for a vehicular navigation solution using low cost MEMS-based inertial sensors during GPS outages.

## Figures and Tables

**Table 1.** Crossbow IMU specifications.

**Specifications**	**IMU 300CC-100**

Update Rate	>100 Hz
	***Gyroscope***
Range	±100 deg/s
Bias	<±2.0 deg/s
Scale Factor	<1%
Angle Random Walk	<2.25 deg/ $\sqrt{\mathrm{hr}}$
	***Accelerometer***
Range	±2 g
Bias	<±30 mg
Scale Factor	<1%
Velocity Random Walk	<0.15 m/s/ $\sqrt{\mathrm{hr}}$
Linearity	<1%

**Table 2.** Maximum position error during the 10 simulated outages for different numbers of visible satellites for the first trajectory.

**Outage No.**	**Maximum Position Error (m)**							
	**PF 3 Sat**	**KF 3 Sat**	**PF 2 Sat**	**KF 2 Sat**	**PF 1 Sat**	**KF 1 Sat**	**PF 0 Sat**	**KF 0 Sat**

1	9.41	6.61	9.73	6.43	19.72	25.46	20.58	25.24
2	5.92	11.77	12.18	22.96	12.83	25.89	11.90	25.22
3	5.14	18.10	5.79	22.23	8.55	25.76	6.05	28.21
4	5.01	8.60	5.25	32.20	19.41	38.72	14.23	36.76
5	10.04	13.75	13.07	19.42	17.82	56.30	15.50	57.53
6	5.67	9.14	6.74	12.27	6.57	22.29	4.75	22.05
7	4.91	9.39	10.31	10.24	18.49	33.40	18.45	33.59
8	11.44	12.83	13.96	13.81	11.51	14.90	11.60	14.89
9	9.99	31.53	13.53	33.85	12.60	44.87	15.22	47.84
10	12.93	9.73	12.77	14.97	13.63	31.42	14.75	27.72

Average	8.04	13.15	10.33	18.84	14.11	31.90	13.30	31.91

**Table 3.** Maximum position error during the 10 simulated outages for different numbers of visible satellites for the second trajectory.

**Outage No.**	**Maximum Position Error (m)**							
	**PF 3 Sat**	**KF 3 Sat**	**PF 2 Sat**	**KF 2 Sat**	**PF 1 Sat**	**KF 1 Sat**	**PF 0 Sat**	**KF 0 Sat**

1	8.99	18.21	10.18	17.72	9.39	24.40	9.53	24.61
2	10.49	16.68	10.61	20.82	17.17	19.02	16.43	18.48
3	7.92	27.28	11.25	27.18	11.41	45.91	16.07	37.31
4	3.23	8.33	3.40	14.10	8.07	6.53	7.81	8.42
5	2.53	2.16	5.82	7.08	4.13	80.27	11.09	82.39
6	4.98	2.81	2.44	14.08	6.05	27.66	16.77	26.81
7	5.11	5.53	5.82	46.47	5.63	93.99	15.85	85.93
8	5.64	6.02	7.83	35.16	10.26	36.76	22.14	24.21
9	4.56	12.56	5.07	23.38	2.29	24.64	10.93	20.79
10	5.67	33.79	5.62	17.15	12.07	32.34	5.10	21.42

Average	5.91	13.34	6.80	22.31	8.65	39.15	13.17	35.04

**Table 4.** RMS and maximum position errors and RMS azimuth errors for the natural outages whose duration exceeds 100 s.

**Outage No.**	**Outage Dur. (s)**	**Approx. Dist. (m)**	**PF Tightly RMS Pos error (m)**	**PF Tightly Max Pos error (m)**	**PF Hybrid RMS Pos error (m)**	**PF Hybrid Max Pos error (m)**	**PF Tightly RMS azimuth error (degree)**	**PF Hybrid RMS azimuth error (degree)**

1	408	1514.60	52.18	118.95	18.22	33.18	6.20	0.78
2	241	973.60	23.56	53.63	15.12	33.74	4.52	1.25
3	125	652.70	46.74	107.58	29.97	63.22	8.75	5.63
4	115	659.40	25.73	57.87	13.74	23.82	5.76	2.55
5	422	473.40	33.37	62.44	13.31	27.38	13.37	2.12
6	173	433.80	10.60	18.64	5.70	11.74	5.01	2.51
7	190	289.00	14.29	27.67	8.12	12.73	7.43	2.18
8	103	152.60	7.30	17.68	6.88	16.92	13.70	11.56

Average	222	643.64	26.72	58.06	13.88	27.84	8.09	3.57

**Table 5.** RMS error in pitch and roll for the natural outages whose duration exceeds 100 s.

**Outage No.**	**Outage Dur. (s)**	**Approx. Dist. (m)**	**RMS Pitch error (Deg)**	**RMS Roll error (Deg)**

1	408	1514.60	0.70	0.27
2	241	973.60	0.74	0.25
3	125	652.70	0.68	0.26
4	115	659.40	0.93	0.29
5	422	473.40	0.62	0.21
6	173	433.80	0.87	0.34
7	190	289.00	0.84	0.17
8	103	152.60	0.92	0.33

Average	222	643.64	0.79	0.27
